# Estimated prevalence of disability and developmental delay among preschool children in rural Malawi: Findings from “Tikule Limodzi,” a cross‐sectional survey

**DOI:** 10.1111/cch.12741

**Published:** 2020-01-26

**Authors:** Rachel Murphy, Emma Jolley, Paul Lynch, Mika Mankhwazi, Jenipher Mbukwa, Stevens Bechange, Melissa J. Gladstone, Elena Schmidt

**Affiliations:** ^1^ Leonard Cheshire Disability London UK; ^2^ Health and Disability Research Sightsavers Chippenham UK; ^3^ Vision Impairment Centre for Teaching and Research University of Birmingham UK; ^4^ Chancellor College University of Malawi Zomba Malawi; ^5^ Paediatric Neurodisability University of Liverpool UK; ^6^ Strategic Programme Innovation, Development and Research, Sightsavers, Chippenham UK

**Keywords:** child development, developmental delay, disability, early intervention, Malawi, special education

## Abstract

**Background:**

Early childhood development (ECD) is a critical stage in children's lives, influencing future development and social integration. ECD research among children with disability and developmental delay in low‐ and middle‐income countries is limited but crucial to inform planning and delivery of inclusive services. This study is the first to measure and compare the prevalence of disability and developmental delay among children attending preschool centres in rural Malawi.

**Methods:**

A cross‐sectional survey was conducted in 48 preschool centres in Thyolo district, Malawi. Data were collected from parents or guardians of 20 children per centre. Disability was ascertained using the Washington Group/UNICEF Child Functioning Module. Child development was measured using the language and social domains of the Malawi Development Assessment Tool.

**Results:**

A total of 960 children were enrolled; 935 (97.4%) children were assessed for disability and 933 (97.2%) for developmental delay; 100 (10.7%) children were identified as having a disability. The prevalence of disability was higher among children 5+ years (*n* = 60; 29.3%) than children 2–4 years (*n* = 40; 5.5%); 109 of 933 (11.7%) children were classified as having developmental delay, 41 (4.4%) in “language” and 77 (8·3%) in “social” domains.

**Conclusions:**

This study found that disability and developmental delays are common among preschool children in Malawi. It is one of the first to measure disability and delay among children in a preschool setting in Africa.

**Key messages**

- The study highlights that disability and developmental delay are common conditions among children attending preschool in low‐income settings. Preschools must be responsive to include children with a variety of impairments to ensure they can participate.
- The study indicates feasibility in the application of the Washington Group/UNICEF Child Functioning Module and the Malawi Development Assessment Tool to measure prevalence of disability and developmental delays in preschool children in rural sub‐Saharan Africa.
- The study contributes to the “leave no child behind” agenda, specifically Sustainable Development Goal 8, “Quality Education”, and the United Nations Convention on the Rights of Persons with Disabilities.

## 1 INTRODUCTION

The World Health Organization (WHO, 2011) estimates more than 93 million children globally aged 0–14 years (5.1%) live with disabilities. Improvements in under five mortality rates in low‐income settings over the past 15 years have left large numbers of surviving children at greater risk of disability and developmental delay (Scherzer, Chhagan, Kauchali, & Susser, 2012). Children with disabilities can be disadvantaged or marginalized in any setting, but the prevalence of undiagnosed disability among children in low‐income settings may be greater than in high‐income settings as a result of insufficient capacity in health and social support systems to identify and respond to children's needs. Little data exists from low‐income settings on the prevalence, causes, or interventions needed to support children with disabilities and developmental delays, hindering effective responses and evidence‐based policy making (Olusanya, 2011).

Early childhood development (ECD), defined as the time from prenatal period to 8 years of age, is a vital stage of an individual's physical, emotional, and intellectual development (WHO/UNICEF, 2012). During these years, the brain develops more than in any other period of life, laying foundations for one's ability to learn, adapt to change, and ultimately succeed in life (Black et al., 2017). Evidence from the past few decades consistently shows that malnutrition, extreme poverty, chronic infections, and low levels of stimulation in early years negatively affect a child's growth and development and may jeopardize their chances to reach their full potential (Grantham‐McGregor et al., 2007; Lake & Chan, 2015). For children with developmental delays or disabilities, this is a critical time to receive early interventions, protection, and support; in the absence of which, difficulties in functioning can become more severe, leading to long‐lasting marginalization and exclusion (WHO/UNICEF, 2012).

The United Nations Convention on the Rights of Persons with Disabilities, the United Nations Convention on the Rights of the Child, and the Sustainable Development Goals recognize the rights of children with disabilities to develop to their full potential (United Nations, 1989, 2007). They confirm the need for inclusive and equitable quality education with easier access to schooling for children with disabilities (McLinden et al., 2018). Despite this, children with disabilities are often excluded from education and learning opportunities, and disability continues to be a neglected issue in the education sector (Mizunoya, Mitra, & Yamasaki, 2018; WHO, 2011).

Data on how children with disabilities access education in low‐ and middle‐income countries (LMICs) are limited, but available studies show children with disabilities are less likely to enter school and have lower attendance rates and lower transition rates to higher levels of education (Kuper et al., 2014). Where children with disabilities do attend school, they are more likely to be at a lower grade for their age and have a lower quality educational experience than their non‐disabled peers (Mizunoya et al., 2018).

ECD programmes have shown to be instrumental in improving young children's capacity to develop and learn (Black et al., 2017). There is growing evidence that children who receive high‐quality early years interventions gain a wide range of skills, helping them to succeed later in formal education and reducing the risk of long‐term disability‐related consequences, increased poverty, and marginalization (Mizunoya et al., 2018). For children with disabilities, ECD programmes provide vital opportunities for individual needs assessments, design of targeted development plans, and building capacities and support networks for parents (Black et al., 2017). However, there is a dearth of data on participation of children with disabilities in ECD programmes in low‐income settings and no studies assessing the effectiveness of interventions to promote inclusion of such children in ECD services, making planning and delivery of more inclusive ECD services at scale difficult (Douglas et al., 2012; World Bank, 2015).

This paper reports the results of a survey that measured the prevalence of disability and developmental delay in children aged 2 years and over attending preschool community‐based childcare centres (CBCCs) in Thyolo district in rural Malawi.

## 2 METHODS

### 2.1 Study setting

Malawi is one of the poorest countries in the world, ranked 171 out of 189 countries on the Human Development Index (United Nations, 2018). The 2015/2016 Demographic and Health Survey Malawi Housing and Population Census estimated disability prevalence among children aged 2–9 years to be 29% (National Statistical Office [Malawi] and ICF, 2017). Malawi launched its National ECD policy in 2018, indicating commitment towards the promotion of early childhood education in the country. However, the Malawian government struggles to implement these laws and policies due to the lack of capacity and resources (MacDonnell Chilemba, 2013).

Malawi was one of the first African countries to set up a network of early childhood centres, CBCCs, to provide care and education to preschool children (Ozler, Karol, Mcconnell, Neuman, & Eduardo, 2016). Over 11,000 CBCCs now exist across Malawi and run by approximately 32,361 community volunteers (Neuman, McConnell, & Kholowa, 2014). The recent government Growth and Development Strategy addresses the need for integrated ECD and aims to increase the number of CBCCs and children with disabilities attending CBCCs (The Ministry of Economic Planning and Development, 2017). At present, there is little evidence on how they support children with disabilities or special needs who attend (Munthali & Silo, 2014).

### 2.2 Study design

Tikule Limodzi (“Let's grow together”) was a 3‐year (2015–2018) mixed‐method study that aimed to explore ways of developing the skills of caregivers to support children attending CBCCs through the use of inclusive strategies and resources (McLinden et al., 2018). This paper presents the findings from the pre‐intervention baseline survey of a cluster‐randomized trial that measured the impact of an inclusive caregiver training package on a variety of child outcomes, specifically the language and social development of children aged 2 years and over attending CBCCs (McLinden et al., 2018). The data presented here were collected between December 2016 and May 2017.

### 2.3 Sampling

The sample size was calculated to detect a 10% change in the proportion of children, whose developmental age is equal to their biological age (expected increase from 70% to 80%) and based on the 95% confidence interval, 80% power, 10% non‐response, and 50% variation between the clusters (Hayes & Bennett, 1999). In total, 960 children, 480 in each arm, were required.

A two‐stage sampling approach was used. The CBCCs were selected from an unpublished sampling frame compiled by the survey team based on the CBCC records available to the District Social Welfare Office. The sampling frame was refined to include only the CBCCs in Thyolo district, which met the following inclusion criteria: CBCCs that had not participated in an earlier study funded by the World Bank (World Bank, 2015); CBCCs with a feeding programme; CBCCs with more than 20 children registered and regularly attending; CBCCs with a minimum of two caregivers; and CBCCs with a minimum infrastructure (e.g., permanent location and water supply). The CBCCs that did not meet the above criteria were excluded. Of the remaining CBCCs, 48 were randomly selected for inclusion in the study.

The second stage involved selection of 20 children per CBCC. The children were randomly selected on the day the data collection team visited the CBCC using a list of all children aged over 2 years who had been registered for at least 6 months and attended the centre regularly (at least 4–5 times a month). Written consent was recorded for all parents/guardians and was witnessed in the case of them being illiterate or aged under 18 years.

### 2.4 Study tools

The two tools used to measure disability and developmental delay were among a suite of assessments collected in the survey.

#### 2.4.1 Disability measurement

The Washington Group/UNICEF Child Functioning Module (CFM) was used to measure functional difficulty in this study (Cappa et al., 2018; Loeb, Cappa, Crialesi, & de Palma, 2017). The CFM assesses functional difficulties in children across a number of domains including vision, hearing, mobility, communication/comprehension, learning, emotions, and playing to identify children who are at greater risk than other children of experiencing limited participation in an unaccommodating environment. The tool has been validated for use with children in two age groups: children aged 2–4 years (16 questions) and children aged 5–17 years (24 questions). The tools were administered using structured face‐to‐face interviews with the parent or caregiver.

Disability was classified using the recommended cut‐off by the Washington Group of “a lot of difficulty” or “cannot do at all” in any one of the domains, “daily” for anxiety and depression, “more” or “a lot more” for controlling behaviour for children aged 2–4 years, and “a lot more” for controlling behaviour for children aged 5+ years.

A second measure, “severe disability,” was constructed by using a cut‐off of “cannot do at all,” “daily” for anxiety and depression, and “a lot more” for controlling behaviour.

Members of the research team translated the questions into the local language, Chichewa, and tested them using backwards and forwards translations.

#### 2.4.2 Developmental assessment tool

The Malawi Development Assessment Tool (MDAT) was used to assess child development (Gladstone et al., 2010). The tool uses culturally appropriate age‐standardized developmental milestones created in Malawi. This study assessed two of the four MDAT domains: “language” and “social,” which were assessed and scored “pass,” “fail,” or “did before/not sure.” The score in each domain was defined as the number of tasks the child completed until the point that the child failed six consecutive tasks. For tasks that could not be assessed, the weighted score was defined as the proportion of the tasks that could be completed, scaled to a total score of 34 for each domain. The calculated score was compared against a reference range for their biological age. Developmental delay in either domain was defined as a child of a given age scoring lower than the 2.5th centile of the reference group of children of the same age (a *Z* score of less than −1.96). The MDAT was already available for use in Chichewa.

### 2.5 Data collection and analysis

Five data collectors were recruited locally and completed a 5‐day training on the tools, their application, child safeguarding issues, and use of mobile devices for data collection. The data collectors also completed an inter‐rater reliability test for the MDAT; all achieved above 90%.

Data were collected using password protected smartphone devices. All data were uploaded to a centrally managed server and backed up by the technical team daily. Data were cleaned and analysed using STATA 14.0.

## 3 RESULTS

### 3.1 Characteristics of the sample

Out of 960 children sampled, 935 (97.4%) were assessed for disability and 933 (97.2%) for developmental delay (language and social domains).

Fifty‐five per cent of screened children were girls (*n* = 517 and *n* = 508). The age of children ranged from 2–10 years with a median age of 4 years (IQR 3–4 years). Seventy‐eight per cent of children were aged 2–4 years (Table 1).

**Table 1:** Characteristics of the children assessed using each tool

Variable	CFM (%)	MDAT (%)
Total	935 (100)	933 (100)
Sex		
Male	418 (44.7)	425 (45.6)
Female	517 (55.3)	508 (54.5)
Age		
2–4 years	730 (78.1)	726 (77.8)
5+ years	205 (21.9)	207 (22.2)

Abbreviations: CFM, Child Functioning Module; MDAT, Malawi Development Assessment Tool.

### 3.2 Prevalence of disability

Out of 935 children, 100 (10.7%) were classified as having a moderate disability and 33 children (3.5%) were classified as having a severe disability.

The prevalence of both moderate and severe disability was slightly higher among boys (*n* = 48, 11.5% and *n* = 17, 4.1%) than girls (*n* = 52, 10.1%; *n* = 16, 3.1%), but the differences were not statistically significant.

The prevalence of moderate disability was higher among children aged 5+ years (*n* = 60, 29.3%) compared with those aged 2–4 years (*n* = 40, 5.5%). Severe disability was also higher among older children (*n* = 23 [11.2%] vs. *n* = 10 [1.4%]). After adjustment for sex, children aged 5+ years were seven times more likely to have a disability than children aged 2–4 years (Table 2).

**Table 2:** Prevalence of disability by sex and age of children

Variable		Prevalence of functional difficulty, *n* (%)	Adjusted OR (95% CI, *p* value)
Total (*n* = 935)		100 (10.7)	
Sex	Male (*n* = 418)	48 (11.5)	Ref
	Female (*n* = 517)	52 (10.1)	0.83 [0.54, 1.29], *p* = .4081^a^
Age	2–4 years (*n* = 730)	40 (5.5)	Ref
	5+ years (*n* = 205)	60 (29.3)	7.17 [4.62, 11.11], *p* < .0012^b^

Abbreviations: CI, confidence interval; OR, odds ratio.

a Adjusted for age.

b Adjusted for sex.

The number of children with disability varied between the CBCCs with as many as eight out of 20 sampled children having a disability in one CBCC and no children with disabilities in six of the 48 CBCCs.

### 3.3 Functional domains

**Figure 1:**
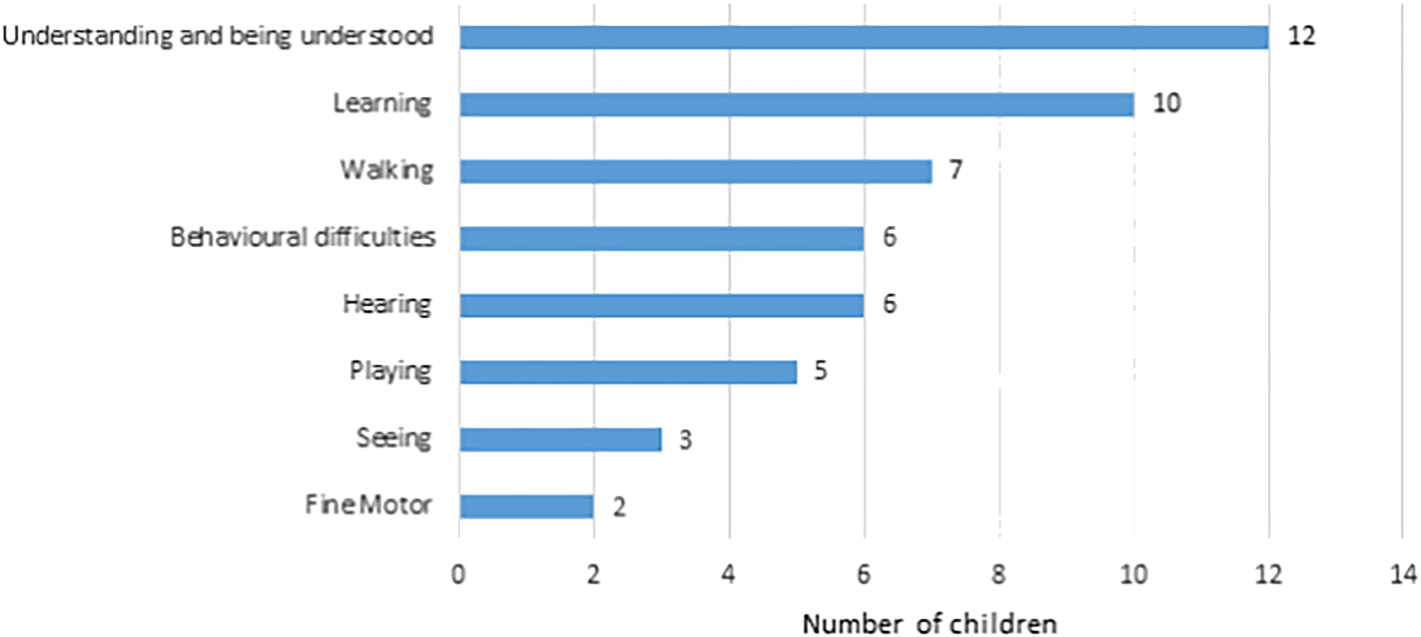
Distribution of difficulties in functional domains among children aged 2–4 years [Colour figure can be viewed at http://wileyonlinelibrary.com]

Among 40 children aged 2–4 years with disabilities, the most commonly reported functional difficulties were “understanding/being understood” and “learning,” followed by “walking,” “hearing,” and “behavioural” difficulties (Table 3 and Figure 1).

**Table 3:** Prevalence of difficulties reported in functional domains in children aged 2–4 and 5+ years

Functional domain	Children aged 2–4 years (*n* = 730)		Children aged 5+ years (*n* = 205)	
	*n*	%	*n*	%
Seeing	3	0.4	5	2.4
Hearing	6	0.8	3	1.5
Walking	7	1.0	6	2.9
Fine motor	2	0.3	n/a	n/a
Understanding and being understood	12	1.6	10	4.9
Learning	10	1.4	6	2.9
Playing	5	0.7	n/a	n/a
Behavioural difficulties	6	0.8	15	7.3
Self‐care	n/a	n/a	7	3.4
Remembering	n/a	n/a	13	6.3
Concentration	n/a	n/a	4	2.0
Accepting change	n/a	n/a	11	5.4
Making friends	n/a	n/a	5	2.4
Anxiety	n/a	n/a	19	9.3
Depression	n/a	n/a	10	4.9

*Note.* Some children had difficulties in multiple domains.

Abbreviation: n/a, not applicable.

Nine out of the 40 children (22.5%) had difficulties in more than one domain; seven experienced difficulties in two domains, and two experienced difficulties in three of the eight domains. Six children were wearing glasses, six children had a hearing aid, and 12 children reported using equipment to help with walking.

**Figure 2:**
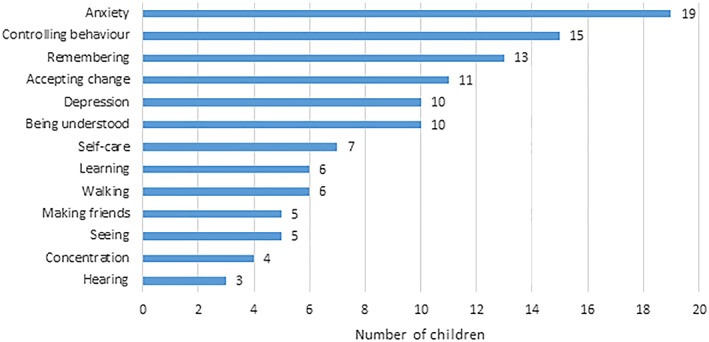
Distribution of difficulties in functional domains among children aged 5+ years [Colour figure can be viewed at http://wileyonlinelibrary.com]

Among the 60 children aged 5+ years identified as having a disability, the most common functional difficulties were anxiety, behavioural problems, remembering, accepting change, understanding/being understood, and depression (Table 3 and Figure 2). Twenty‐three of the 60 children (38.3%) experienced difficulties across multiple domains. Fifteen children had difficulties in two domains, two children in three domains, one child in four domains, one child in five domains, two children in seven domains, and two children in nine of the 13 domains. One child was wearing glasses, three had a hearing aid, and four used equipment to help them walk. One of these four children reported that they were unable to walk without this equipment; three experienced some difficulty when walking without their equipment.

### 3.4 Prevalence of developmental delay

Out of 933 children screened for developmental delay using the language and social domains of the MDAT tool, 109 (11.7%) were classified as having a developmental delay in at least one domain. Prevalence of developmental delay in both domains was higher among boys (13.6% compared with 10%) and among children aged 5+ years (17.0%) compared with 2‐ to 4‐year‐olds (10.2%).

Forty‐one children experienced delay in the language domain (4.4%) with little difference between boys (4.5%) and girls (4.3%) but slightly higher prevalence among children aged 2–4 years (4.5%) than those aged 5+ years (3.9%). Seventy‐seven children experienced delay in the social domain (8.3%). The prevalence was higher among boys (10.6%) than girls (6.3%) and among children 5+ years (15.2%) than those aged 2–4 years (6.3%). Nine (1.0%) children experienced delays in both domains.

Sensitivity analysis using a cut‐off point below the fifth centile of the standardized reference range resulted in an increased prevalence of developmental delay to 14.9% (*n* = 139); the prevalence of language‐related delays increased to 4.9% (*n* = 46); the prevalence of social delays increased to 11.3% (*n* = 105).

### 3.5 Relationship between disability and developmental delay

Nine hundred thirty children responded to both the CFM and MDAT. Among 100 children categorized as having disability, 98 also had MDAT results. Thirty‐two (32.7%) of these 98 children had a development delay, the majority (28 children) having delays in the social domain; 11 children with disabilities had language‐related delays, whereas seven children (7.1%) had delays in both domains. Having a disability was strongly associated with having a developmental delay; children with disabilities were 4.8 times more likely to have a developmental delay than children without disabilities (odds ratio 4.75; *p* value < .0001).

No associations between delays and specific domains of impairment were observed (Table 4).

**Table 4:** Distribution of correspondence between identified delays and domains in which difficulties were reported

Functional difficulty domain	Children aged 2–4 years (*n* = 725)			Children aged 5+ years (*n* = 205)		
	Language delay	Social delay	No delay	Language delay	Social delay	No delay
Seeing	0	2	1	0	1	4
Hearing	2	0	4	0	2	1
Walking	0	0	6	2	3	2
Fine motor	0	1	1	n/a	n/a	n/a
Understanding and being understood	4	4	5	4	4	6
Learning	2	4	5	2	2	4
Playing	1	2	3	n/a	n/a	n/a
Behavioural difficulties	1	1	5	2	8	7
Self‐care	n/a	n/a	n/a	1	3	4
Remembering	n/a	n/a	n/a	2	2	11
Concentration	n/a	n/a	n/a	2	2	2
Accepting change	n/a	n/a	n/a	2	3	8
Making friends	n/a	n/a	n/a	3	3	2
Anxiety	n/a	n/a	n/a	2	5	14
Depression	n/a	n/a	n/a	2	4	6

Abbreviation: n/a, not applicable.

## 4 DISCUSSION

This study is one of the first in an LMIC to assess the prevalence of disability and development delay among preschool children. The study found one in 10 children attending CBCCs had a disability and 3.5% had a severe disability. No differences were observed between sexes, but older children were more likely to have a disability than younger children.

Over 11% of children had a delay in at least one of the two domains, and the prevalence of developmental delay was higher among older children. Delays in the social domain were more common than language delays, and the age and sex differences observed were largely due to the differences in the social domain. We also found a high correlation between delay in language and social development and disability: children with disabilities were 4.8 times more likely to have a developmental delay than children without disabilities. Similar conclusions were made during the validation of the CFM (Cappa et al., 2018).

Data on disability and developmental delay in young children are difficult to compare across contexts; few studies available use different definitions and measurement tools. The CFM and MDAT are both relatively new tools, and there are only a handful of studies that used either in a field setting.

The Global Burden of Diseases, Injuries, and Risk Factors Study 2016 defined developmental disabilities as a group of conditions resulting from impairments that affect a child's physical, learning, or behavioural functioning (Olusanya et al., 2018). Despite the acknowledged limitations of the available primary data, the study estimated a prevalence of developmental disabilities in children under 5 to be 8.4% or 52.9 million with 94.9% (50.2 million) of these children living in LMICs. Our findings are in line with the estimates made in the Global Burden of Diseases study, suggesting that both functional difficulties and developmental delays are common among preschool children in this part of Malawi. A number of social factors such as poverty, malnutrition, lack of care, and stimulation are likely to be main risks for both functional difficulties and developmental delays in this setting.

This study contributes to the growing evidence calling for more intensive early childhood interventions targeting young children in this and similar settings. This study also shows that the application of the CFM and MDAT is feasible and can be used to measure prevalence of disability and development delays in preschool children in rural parts of sub‐Saharan Africa.

A few methodological issues need to be considered when interpreting the findings of this study. We did not collect anthropometric measures, and MDAT findings are not adjusted for these variables. The findings on disability and developmental delay were not adjusted for confounding factors other than age and sex; other factors, such as socio‐economic status of the household, household crop/food availability, religion, and parental education, also affect child development and need to be measured in future research.

This study makes a significant contribution to the literature on disability and developmental delay in preschool children in LMICs. It indicates the numbers, types, and severity of impairments experienced by young children in this setting and provides data upon which more inclusive policies and practices to support their educational and social development can be developed.

## CONFLICT OF INTEREST

The authors declare no competing interests.

## ETHICAL STATEMENT

Ethical approval for this study was obtained from the National Committee on Research in the Social Sciences and Humanities, National Commission for Science and Technology, Malawi (P.02/16/83), and the University of Birmingham Ethics Committee, UK (ERN_15‐0048).
